# Increase in leaf temperature opens stomata and decouples net photosynthesis from stomatal conductance in *Pinus taeda* and *Populus deltoides x nigra*

**DOI:** 10.1093/jxb/erx052

**Published:** 2017-03-06

**Authors:** Josef Urban, Miles W. Ingwers, Mary Anne McGuire, Robert O. Teskey

**Affiliations:** 1 Department of Forest Botany, Dendrology and Geobiocenology, Mendel University in Brno, Brno, Czech Republic; 2 Siberian Federal University, Krasnoyarsk, Russia; 3 Institute of Plant Breeding, Genetics and Genomics, University of Georgia, Athens, GA, USA; 4 Daniel B. Warnell School of Forestry and Natural Resources, University of Georgia, Athens, GA, USA

**Keywords:** Ball–Berry model, elevated temperature, evaporative cooling, global change, heat waves, stomatal conductance.

## Abstract

The effect of temperature on stomatal conductance (*g*_s_) and corresponding gas exchange parameters was studied in two tree species with contrasting leaf anatomy and ecophysiology—a broadleaf angiosperm, *Populus deltoides x nigra* (poplar), and a needle-leaf gymnosperm, *Pinus taeda* (loblolly pine). Experiments were conducted in growth chambers across a leaf temperature range of 19–48°C. Manipulations of temperature were done in well-watered and drought soil conditions and under ambient (400 ppm) and elevated (800 ppm) air CO_2_ concentrations. Increases in leaf temperature caused stomatal opening at both ambient and elevated [CO_2_]. The *g*_s_ increased by 42% in poplar and by 40% in loblolly pine when leaf temperature increased from 30°C to 40°C at a vapour pressure difference of 1 kPa. Stomatal limitation to photosynthesis decreased in elevated temperature in loblolly pine but not in poplar. The ratio of net photosynthesis to *g*_s_ depended on leaf temperature, especially at high temperatures. Evaporative cooling of transpiring leaves resulted in reductions in leaf temperature up to 9°C in well-watered poplar but only 1°C in drought-stressed poplar and in loblolly pine. As global mean temperatures rise and temperature extremes become more frequent and severe, understanding the effect of temperature on *g*_s_, and modelling that relationship, will become increasingly important.

## Highlight

Stomatal conductance of two species (a broadleaf and a conifer) increased with increasing temperature. This response was independent of carbon metabolism, plant water status, or vapour pressure difference.

## Introduction

Plant stomata play a key role in water and carbon cycles. On average, plant transpiration accounts for 61% of global evapotranspiration (Schlesinger and Jasechko, 2014). In other words, most water moving from terrestrial ecosystems into the atmosphere passes through plants and the precise amount is regulated by stomata. At the same time, stomatal conductance (*g*_s_) is a key factor determining the rate of net photosynthesis (*A*) and, therefore, the global carbon cycle and plant carbon metabolism. As a result, stomatal regulation is one of the main factors which determine local growth and survival of plants and global cycles of mass and energy. Stomatal conductance is so important that it has become central to many models from the leaf level (Ball *et al.*, 1987; Leuning *et al.*, 1995; Jarvis and Davies, 1998; Tuzet *et al.*, 2003), to the tree- and forest-stand level (Mirfenderesgi *et al.*, 2016; Xu *et al.*, 2016), and even up to the global level (Niyogi *et al.*, 2009; Berry, 2012; Verhoef and Egea, 2014). However, the conditions in which plants grow are changing and we still do not know enough about plant stomatal regulation to predict future stomatal responses of plant species and their effects at ecosystem and global scales (Lin *et al.*, 2015).

Temperature is one of the most variable factors in the environment and it affects many plant physiological processes, yet little is known about its effect on *g*_s_, especially at high temperatures (Teskey *et al.*, 2015). Historically, temperatures over 40°C have been recorded in many places in North America. It has been predicted that mean maximum summer temperatures will increase 5°C in the eastern US within this century (Lynn *et al.*, 2007). Here, we studied effects of temperature on the leaf gas exchange of two North American tree species, *Pinus taeda* (loblolly pine) and *Populus deltoides x nigra* (hybrid poplar). Loblolly pine is native to the south-eastern US where the highest temperatures recorded among the 12 states in the region range from 43 to 49°C, with a mean maximum temperature for all 12 states of 45°C (National Climatic Data Center, 2016). Hybrid poplar is widely planted in the Northern Great Plains, which includes the states of Nebraska, Wyoming, Montana, North Dakota and South Dakota. The highest recorded temperatures in those states range from 46 to 49°C with a mean of 48°C. In addition to increases in mean air temperature (*T*_a_), the frequency of extreme temperatures and the severity of heat waves have also increased, and are likely to increase further (Meehl and Tebaldi, 2004; Perkins *et al.*, 2012). Summertime extreme temperatures associated with prolonged heat waves now impact approximately 10% of land surfaces, up from 1% in the 1960s (Hansen *et al.*, 2012). Over recent decades record-breaking monthly temperature extremes have occurred five times more often than during the late 19th through the mid-20th century (Coumou and Robinson, 2013). Heat waves are usually associated with low precipitation and soil drought (Ciais *et al.*, 2005; Stéfanon *et al.*, 2014). However, the frequency of heat waves during wet periods is also increasing. When temperature and precipitation were compared between the periods of 1951–1977 and 1978–2004, it was apparent that both wet/hot and dry/hot conditions were increasing substantially worldwide (Hao *et al.*, 2013). Effects of the increasing frequency and severity of extreme temperature events on *g*_s_ are largely unknown.

Results of experiments that examined the direct dependence of *g*_s_ on temperature have not been consistent. Previous studies have reported a complete range of responses to increased temperature, including stomatal opening (Schulze *et al.*, 1974; Freeden and Sage, 1999; Lu *et al.*, 2000; Mott and Peak, 2010), no significant response (Teskey *et al.*, 1986; Sage and Sharkey, 1987; Cerasoli *et al.*, 2014; von Caemmerer and Evans, 2015), and stomatal closure (Raven *et al.*, 2005; Weston and Bauerle, 2007; Lahr *et al.*, 2015). A peaked response with maximum *g*_s_ at 20°C (Way *et al.*, 2011) or more complex responses with one peak between 20 and 30°C and an additional increase at extremely high temperatures (Slot *et al.*, 2016) have also been described. One possible explanation for these inconsistent results is that to isolate the direct effect of temperature on *g*_s_ requires a well-controlled environment, which is often hard to achieve, particularly with respect to vapour pressure difference (VPD). In addition, differences in sensitivity to heat are likely related to species, whether plants were grown in the laboratory or in the field, and the range of measurement temperature (Slot *et al.*, 2016).

It has been well established that plants regulate rates of transpiration and photosynthesis in parallel, maintaining a balance between *g*_s_ and *A* (Lawson *et al.*, 2011). Therefore, the effect of temperature on stomata is often considered to be indirect, through VPD, transpiration, leaf water potential, or the effect of temperature on photosynthesis or intercellular [CO_2_] (*C*_i_). This parallel regulation results in the conservation *C*_i_ at a given atmospheric [CO_2_] (*C*_a_) and a close correspondence between *g*_s_ and *A* (Wong *et al.*, 1979; Hetherington and Woodward, 2003). The latter relationship has been central to several models of stomatal control of photosynthesis (Farquhar and Wong, 1984; Ball *et al.*, 1987; Leuning, 1995; Buckley *et al.*, 2003), which assume that the ratio of *g*_s_ correlates with *A* over a wide range of environmental conditions. However, some studies indicated that this relationship was decoupled under extreme temperature during heat waves, such that *A* decreased, but *g*_s_ did not. For example, during an imposed heat wave in which daily maximum *T*_a_ ranged from 47 to 53°C and VPD ranged from 6 to 8 kPa, *Pinus taeda* and *Quercus rubra* seedlings exhibited progressively lower *A* on each day of the heat wave but almost no change in *g*_s_ (Ameye *et al.*, 2012). Similarly, *g*_s_ of *Acer rubrum* changed very little across a temperature range of 35 to 48°C (Weston and Bauerle, 2007). In a study of five species, *g*_s_ either increased or did not decline as *T*_a_ increased from 20 to 40°C, even though *A* initially increased from 20 to 30°C and then decreased (von Caemmerer and Evans, 2015). Collectively these studies suggest that the mechanism modulating stomatal aperture may be independent of *A* at higher temperatures. However, because VPD varied with temperature in all of these studies, it could not be determined to what degree the observed changes in *g*_s_ were due to a change in VPD or in the *A*, or were a direct response to temperature.

In this study, we addressed the following questions: (i) What is the direct effect of moderate to high temperature on *g*_s_? (ii) Is the effect of moderate to high temperature on *g*_s_ altered by water stress or *C*_a_? (iii) How does the response of *g*_s_ to temperature link to other related factors such as *A*, *C*_i_, and water status (transpiration, water potential), and how does the correlation between *g*_s_ and *A*, which is crucial to many models, change with temperature? (iv) What is the magnitude of evaporative cooling under extreme temperatures? To answer these questions we performed leaf gas exchange measurements on two contrasting tree species: poplar (*Populus deltoides x nigra*) and loblolly pine (*Pinus taeda*) across a range of temperature and humidity and under well-watered and drought-stress conditions.

## Material and methods

### Growth chambers and tree material

Trees were grown, and measurements conducted, in two walk-in growth chambers (EGC 36, Environmental Growth Chambers, Chagrin Falls, OH, USA) at the University of Georgia campus in Athens, GA, USA. Prior to the start of experimental treatments, the trees were grown in the chambers for 30 days at 26°C/ 23°C (day/night) *T*_a_, 1700/560 Pa (day/night) air VPD, and a daily light period of 13 hours. Photosynthetically active radiation (PAR) in the chambers was 520 μmol m^−2^ s^−1^. Air speed in each chamber was maintained at 1 m s^−1^. During the growth period the *C*_a_ was maintained above 400 µmol mol^−1^ as follows: a CO_2_ sensor (GMM 220, Vaisala, Helsinki, Finland) monitored [CO_2_] in each chamber and controlled a solenoid valve that released CO_2_ from a compressed gas cylinder into the chamber whenever the [CO_2_] fell below the 400 µmol mol^−1^ set point. Although this procedure prevented the [CO_2_] from decreasing below 400 µmol mol^−1^ during periods of active photosynthesis, it did not prevent increases above 400 µmol mol^−1^. To mitigate the build-up of CO_2_ in the chambers, the exterior room windows were fully opened and a large exhaust fan was placed in one window. We estimate that daytime ambient [CO_2_] in the chambers was typically between 400 and 475 µmol mol^−1^.

Measurements were made on clones of two tree species: a poplar (*Populus deltoides x nigra*) clone obtained as cuttings (OP-367, hybridpoplars.com, Glenmoore, PA, USA) and a loblolly pine (*Pinus taeda*) clone from the South Carolina Coastal Plain (Arborgen, Ridgeville, SC, USA). Two-year-old loblolly pine saplings, originally grown in 4-L pots in a greenhouse under natural temperature fluctuations with temperatures commonly reaching ~ 40°C, and poplar cuttings were planted in March 2014 into 15-L pots in a potting medium (Cofer’s Nursery Mix, Cofer’s, Athens, GA, USA). Each pot was fertilized with 40 g of 15-9-12 extended release fertilizer (Osmocote Plus #903286, Scotts-Sierra Horticultural Products, Marysville, OH, USA) and 0.2 g of chelated iron (Sprint 138, Becker Underwood, Ames, IA, USA). Trees were watered daily to full soil water capacity. At the beginning of the experiment, in April 2014, the mean stem height of the poplars was 1.05 m, and the diameter 10 cm above soil was 9.2 mm. The mean height and diameter of the loblolly pines were 1.1 m and 13.9 mm, respectively.

### Gas exchange measurements

Measurements of light-saturated net photosynthesis (*A*_sat_), *g*_s_, rate of transpiration (*E*), and *C*_i_ were made with a portable photosynthesis system equipped with a CO_2_ mixer (LI-6400–20, LiCor Biosciences, Lincoln, NE, USA). Leaf cuvette conditions were set as follows: block temperature was set at ambient (growth chamber) temperature; [CO_2_] was set at either 400 µmol mol^−1^ or 800 µmol mol^−1^, equal to the concentration in the growth chamber; relative humidity was maintained equal to that in the growth chamber; and PAR was set at 1200 µmol m^−2^ s^−1^, resulting in light-saturated photosynthesis and no decline as a result of photorespiration (see figure 2 in Ingwers *et al.* (2016) for the photosynthetic light response curve of loblolly pine trees of the same clone measured in the same growth chambers). Measurements of loblolly pine foliage were made on two fully developed fascicles (six needles total) of the second flush attached to the main stem. The needles were arranged in the cuvette on a flat plane with equal spacing between needles to maximize light interception. After the gas exchange measurement, the widths of each of three sides of the needles were measured with a scale loupe and used to calculate the foliage area in the cuvette. For poplar, measurements were made on approximately the 30th leaf from the top of the plant. Gas exchange measurements were performed on six trees of each species (*n*=6). Gas exchange results were calculated on a total surface area basis for loblolly pine and a one-sided surface area basis for poplar.

### Experimental setup

#### Responses to changes in temperature and VPD under various [CO_2_] and soil moisture

To determine stomatal responses to temperature and VPD, *T*_a_ in the growth chamber was controlled at 20, 30, 40, or 49°C and relative humidity was changed from approximately 30 to 80% at each temperature. The sequence of the temperature changes was chosen randomly and individual trees were excluded from further measurements after they had been subjected to 49°C. Six trees were allowed to acclimate for at least 45 minutes after each change in environmental conditions. At every measurement, *g*_s_, *A*, *E*, and *C*_i_ were recorded. To ensure high water availability, during the measurement period the base of each pot was placed in a 5-cm-tall container that was kept full of water. Pre-light water potential (Ψ_P_) and water potential at varying *T*_a_ and VPD in the light were measured on foliage using a pressure chamber (model 700, PMS Instrument, Albany, OR, USA). Mean Ψ_P_ was −0.28 ± 0.02 and −0.13 ± 0.02 MPa (mean ± standard error) for loblolly pine and poplar, respectively. Measurements were conducted under ambient [CO_2_] (400 µmol mol^−1^) and elevated [CO_2_] (800 µmol mol^−1^). For measurements under elevated [CO_2_], the [CO_2_] was increased in the growth chamber to 800 µmol mol^−1^ as described above by reprogramming the set point of the CO_2_ sensor. The plants were allowed to equilibrate to elevated [CO_2_] for 24 hours prior to measurements.

In a subsequent experiment the effect of soil water deficit on the stomatal response to temperature was investigated. After withholding water for 5 days, the mean Ψ_P_ of the poplar plants was −0.81 ± 0.10 MPa. After withholding water for 12 days, the mean Ψ_P_ of the loblolly pine plants was −0.97 ± 0.06 MPa. On those days, measurements were made using the same combinations of temperature and humidity as in the first experiment. The effect of water deficit was studied only at ambient [CO_2_]. The first experiment and this experiment were conducted on different trees (*n*=6 for each experiment).

#### Effect of *C*_i_ on *A*_sat_ at various temperatures

Under well-watered conditions, *A*/*C*_i_ curves were measured in the growth chamber on six trees of each species. The VPD was held constant at 1.2 kPa at a leaf temperature (*T*_l_) of 20°C and 3.5 kPa at a *T*_l_ of 30°C and 40°C both in the growth chamber and the cuvette. PAR in the cuvette was set at 1200 µmol m^−2^ s^−1^. The [CO_2_] in the cuvette was manipulated from 50 to 100 µmol mol^−1^ and then in 100 µmol mol^−1^ steps to 1800 µmol mol^−1^. The *A*/*C*_i_ Curve Fitting Utility, version 1.1 (Long and Bernacchi, 2003) was used to determine the maximum rate of Rubisco carboxylation (*V*c_max_, μmol m^−2^ s^−1^), maximum rate of photosynthetic electron transport (*J*_max_, μmol m^−2^ s^−1^), maximum rate of triose-phosphate utilization (*V*_TPU_, μmol m^−2^ s^−1^), and day respiration in the absence of mitochondrial respiration (*R*_d_^*^, μmol m^−2^ s^−1^).

Stomatal limitation to photosynthesis (*L*_s_) was estimated at [CO_2_] 400 µmol mol^−1^ from fitted curves using the equation:

$$L_{s}=\frac{A_{0}-A_{sat}}{A_{0}}$$ (1)

where *A*_0_ is the light-saturated net photosynthesis rate that would occur at infinite *g*_s_ (Farquhar and Sharkey, 1982).

#### Cooling effect

Under lighted conditions, the cooling effect of transpiration was estimated as the difference between the temperature of normal transpiring foliage and foliage greased with petroleum jelly to prevent transpiration (Jones *et al.*, 2002) at the same position on the plant. Leaves and needles were chosen for this comparison at a position on the plant close to the point where gas exchange was measured. *T*_l_ was measured with an infrared thermometer (Model 561, Fluke, Everett, WA, USA) with emissivity set to 0.97.

#### Statistical analysis

Prior to the analyses, the normality of data was determined using the Shapiro–Wilk test. We used linear and non-linear multiregression analysis to describe the dependence of *g*_s_ on external factors (i.e. *T*_l_, VPD). A least-squares regression was used to fit the 3D models to the data. Models used to fit data are listed in Supplementary Table S1 (available at *JXB* online). An F-test was used to test significance of model parameters. Analysis of the generalized linear model was used to test for differences among independent variables and a dependent variable when VPD was a continuous predictor. Tests were performed at α=0.05. Most statistical analyses were performed using SigmaPlot 12.5 software (Systat, San Jose, CA, USA) with the exception of the generalized linear model analysis, which was done in Statistica 12 (StatSoft, Tulsa, OK, USA).

## Results

### Responses of *g*_s_, *E*, and *A*_sat_ to *T*_l_ and VPD

**Fig. 1.**
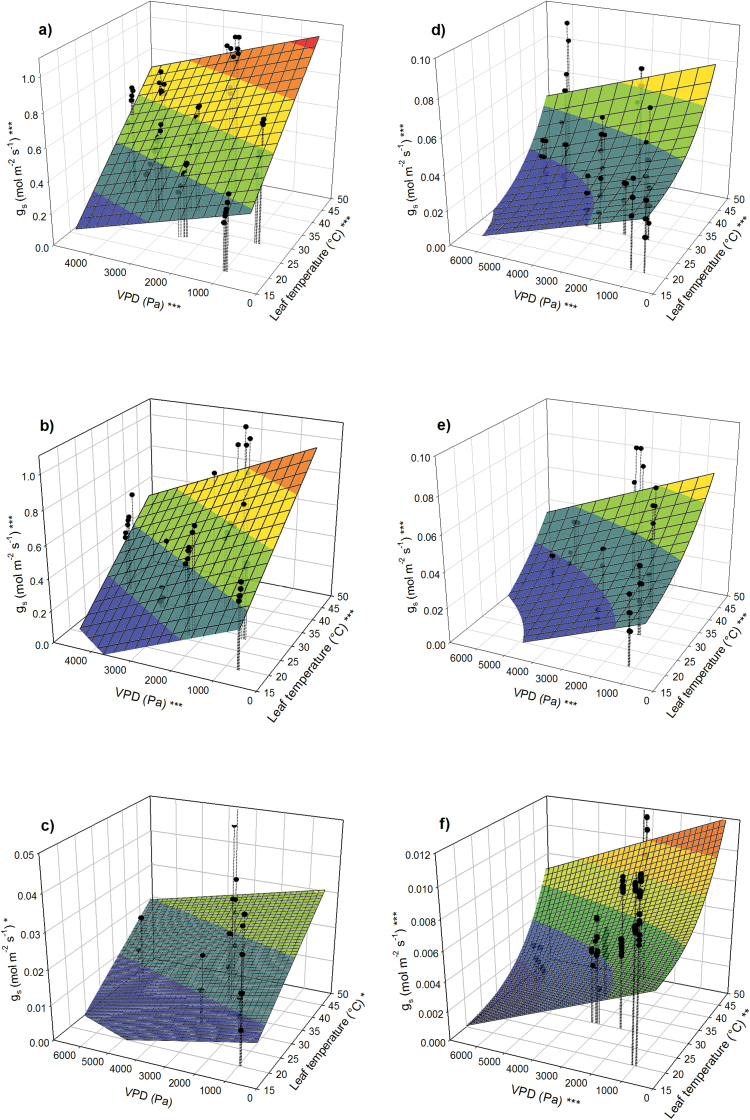
Stomatal conductance (*g*_s_) of poplar (left panels) and loblolly pine (right panels) and its dependence on *T*_l_ and VPD. Plants were measured in high soil moisture conditions and (**a**, **d**) ambient [CO_2_] or (**b**, **e**) elevated [CO_2_]. (**c**, **f**) Measurements made on drought-stressed trees at ambient [CO_2_]. Linear regression was used to fit the data for poplar and non-linear regression was used for loblolly pine. Asterisks at the *z*-axis label indicate overall significance of the model; asterisks at the *x*-and *y*-axes indicate significance of the respective parameters (*P < 0.05; **P < 0.01; ***P < 0.001).

The *g*_s_ increased with increasing *T*_l_ and *T*_a_ in both species in all tested environmental conditions (Fig. 1 and Supplementary Fig. S1, available at *JXB* online). Under unlimited soil water availability and a VPD of 1 kPa and [CO_2_] of 400 μmol mol^−1^, an increase in *T*_l_ from 30 to 40°C led to an increase in *g*_s_ of 42% in poplar and 40% in loblolly pine (Fig. 1a, d; Supplementary Table S1; *P* < 0.001). The rate of increase in *g*_s_ with temperature was linear in poplar, but *g*_s_ increased more at high than at low *T*_l_ in loblolly pine. Increasing the [CO_2_] from 400 to 800 μmol mol^−1^ caused partial stomatal closure, which was more pronounced in poplar (mean decrease of 21% at VPD 3.5 kPa, *P* < 0.001) than in loblolly pine (mean decrease of 12% at the same VPD, *P* = 0.030). However, similar to results in ambient [CO_2_], *g*_s_ increased with increasing *T*_l_ in both species under elevated [CO_2_] (Fig. 1b, e; *P* < 0.001). Soil water deficit significantly reduced *g*_s_ in both species, but more so in poplar than pine (Fig. 1c, f; *P* < 0.001). Even though *g*_s_ was reduced in drought conditions, *g*_s_ of both species still increased with increasing *T*_l_ (*P* = 0.040 for poplar and *P* < 0.001 for loblolly pine).

**Fig. 2.**
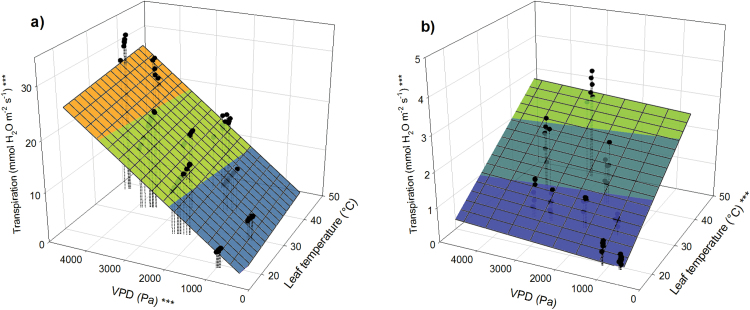
Response of *E* to VPD in (**a**) poplar and (**b**) loblolly pine at varying *T*_l_ and VPD. Asterisks at the *z*-axis label indicate overall significance of the model; asterisks at the *x*- and *y*-axes indicate significance of the respective parameters (*P < 0.05; **P < 0.01; ***P < 0.001).

Transpiration (*E*) increased significantly with increasing *T*_l_ (and *T*_a_) or VPD in both species under unlimited soil water availability and ambient [CO_2_] (Fig. 2a, b and Supplementary Fig. S2a, b, available at *JXB* online). However, the relationships between *E* and environmental variables differed substantially between poplar and loblolly pine. Transpiration of poplar increased with VPD (*P* < 0.001) but not with *T*_l_ (*P* = 0.06). Conversely, in loblolly pine, *E* increased only with *T*_l_ (*P* < 0.001) but not with VPD (*P* = 0.15).

**Fig. 3.**
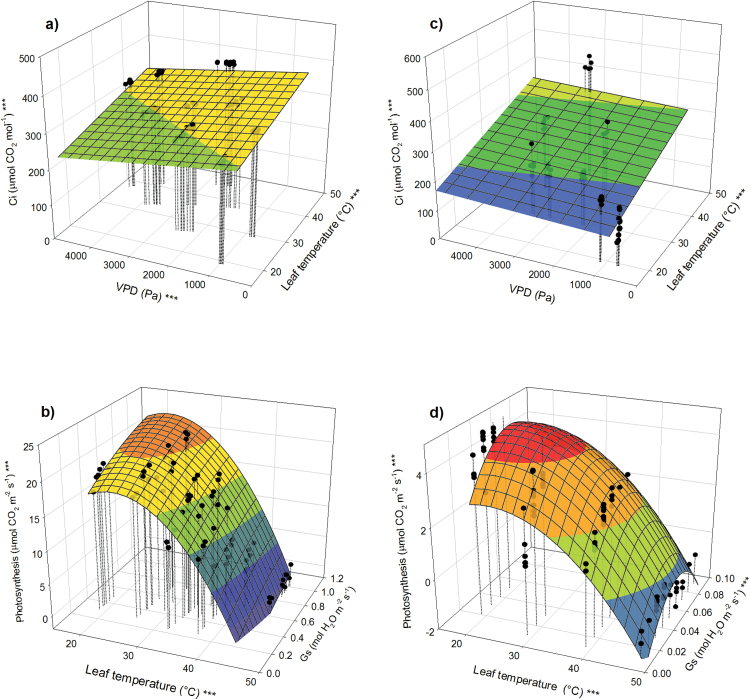
(**a**, **c**) Relationship between *C*_i_, *T*_l_, and VPD for poplar (left panels) and loblolly pine (right panels). (**b**, **d**) Relationship between *A*_sat_, *T*_l_, and *g*_s_. Asterisks at the *z*-axis label indicate overall significance of the model; asterisks at the *x*- and *y*-axes indicate significance of the respective parameters (*P < 0.05; **P < 0.01; ***P < 0.001).

Under well-watered conditions, *C*_i_ increased with increasing temperature in both species (Fig. 3a, c, *P* < 0.001, and Supplementary Fig. S2c, d). A decrease in *C*_i_ with increasing VPD was observed in poplar (*P* < 0.001) but not in loblolly pine (*P* = 0.15). In addition, the range of *C*_i_ was smaller in poplar than in loblolly pine. *T*_l_ (and *T*_a_) had an effect on *A* in both species (Fig. 3b, d, *P* < 0.001, and Supplementary Fig. S3, available at *JXB* online). In both species, at a given *T*_l_ there was a specific relationship between *A*_sat_ and *g*_s_. However, this relationship between *A*_sat_ and *g*_s_ changed with *T*_l_ (Fig. 3b, d, *P* < 0.001).

### *A*/*C*_i_ curves and *L*_s_ to *A*_sat_ at various *T*_l_

Temperature had a large effect on the parameters of *A/C*_i_ curves in both poplar and loblolly pine (Table 1). Stomata of poplar imposed a smaller limitation on the diffusion of CO_2_ than stomata of loblolly pine. The relative *L*_s_ in poplar did not exceed 20% while in loblolly pine they were between 23 and 78%. *L*_s_ was directly comparable between 30 and 40°C because it was measured at the same VPD. While *L*_s_ in poplar did not change (*P* = 0.21) with a *T*_l_ increase from 30 to 40°C, *L*_s_ in loblolly pine declined under the same temperature increase (*P* < 0.001). The values of parameters related to biochemical processes of photosynthesis (*V*c_max_, *J*_max_, *V*_TPU_, and *R*_d_^*^) consistently increased with *T*_l_ in both species, with the exception of *V*_TPU_ in poplar.

**Table 1.** Parameters related to biochemical processes of photosynthesis in poplar and loblolly pine plants measured at three leaf temperatures

Species	*T*_l_ *(°C*)	*V*c_max_	*J*_max_	*V*_TPU_	*R*_d_^*^	*L*_s_
Poplar	20	66	132	10.05	2.10	0.19
	30	165	151	11.11	1.9	0.16
	40	301	165	11.46	3.25	0.2
*P*-value		**<0.001**	**<0.001**	0.07	**<0.001**	0.21
Loblolly pine	20	21	45	3.62	1.55	0.41
	30	67	71	4.57	2.73	0.78
	40	163	75	4.99	6.52	0.23
*P*-value		**<0.000**	**<0.001**	**<0.001**	**0.011**	**<0.001**

Significant differences between measurements at different temperatures indicated in bold.

### Effect of *E* on *T*_l_

**Fig. 4.**
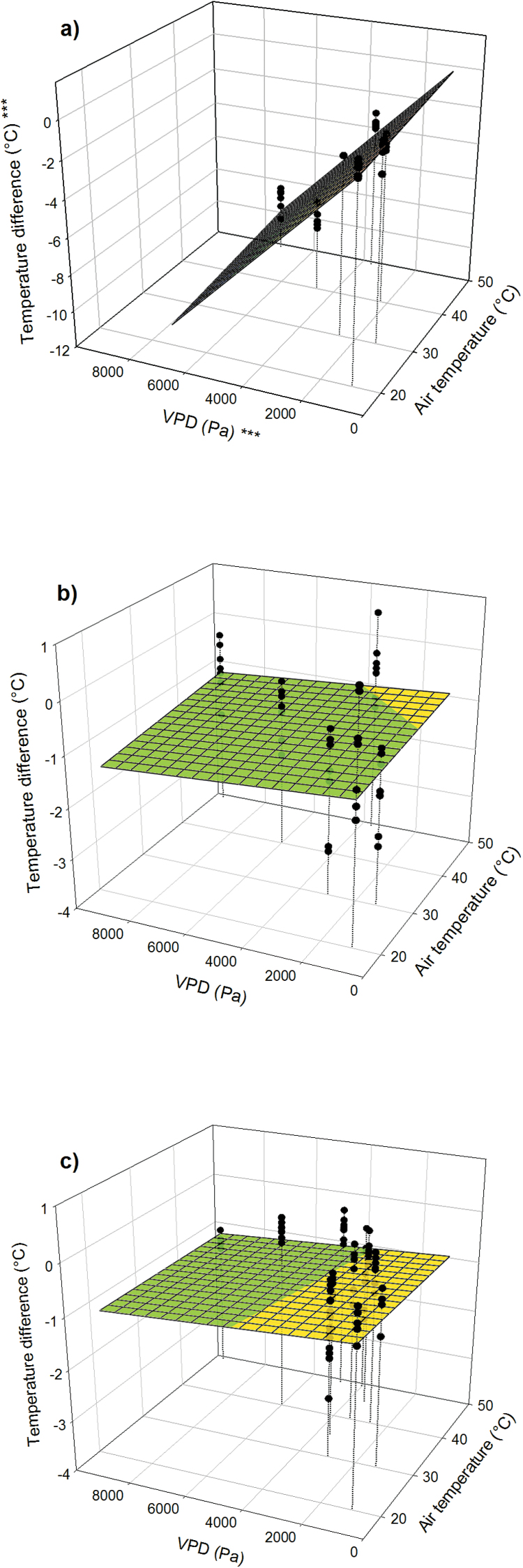
Evaporative cooling effect (temperature difference) of transpiration on (**a**) well-watered poplar, (**b**) drought-stressed poplar, and (**c**) loblolly pine at varying *T*_a_ and VPD. Asterisks at the *z*-axis label indicate overall significance of the model; asterisks at the *x*- and *y*-axes indicate significance of the respective parameters (*P < 0.05; **P < 0.01; ***P < 0.001).

The temperature of transpiring leaves was lower than the temperature of foliage that did not transpire (Fig. 4). The magnitude of the temperature difference in poplars in wet soil reached up to 9.0°C and scaled with VPD (*P* < 0.001) but not with *T*_a_ (Fig. 4a). Transpiring leaves of poplar in dry soil were an average of 1.1°C cooler than non-transpiring leaves (*P* = 0.02) and the magnitude of the cooling effect depended on neither temperature nor VPD (Fig. 4b). In loblolly pine, transpiring needles were an average of 0.9°C cooler than those that did not transpire (*P* = 0.002). There was no effect of soil water availability and the magnitude of the cooling did not depend on temperature or VPD (Fig. 4c).

### Leaf water potential

**Fig. 5.**
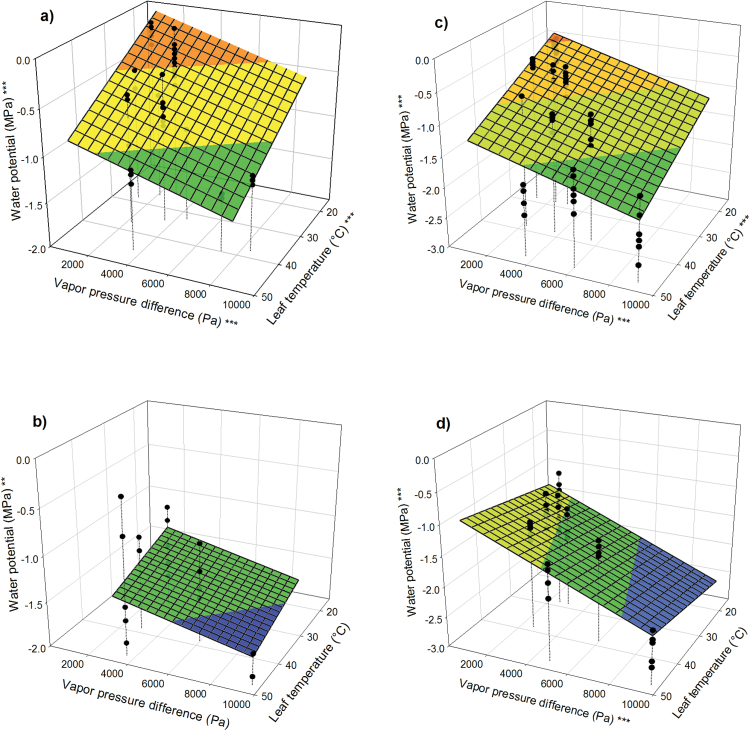
Leaf water potential of (**a**, **b**) poplar and (**c**, **d**) loblolly pine in wet soil (a, c) and dry soil (b, d). Asterisks at the *z*-axis label indicate overall significance of the model; asterisks at the *x*- and *y*-axes indicate significance of the respective parameters (*P < 0.05; **P < 0.01; ***P < 0.001).

Leaf water potential decreased with increasing *T*_l_ and VPD in both species when the soil was wet (Fig. 5a, c). When soil was dry, leaf water potential scaled with both *T*_l_ and VPD in poplar, but in loblolly pine only VPD had an effect on water potential (Fig. 5b, d). At the same *T*_l_ and VPD, poplar maintained higher water potential than loblolly pine.

## Discussion

### Stomatal conductance, stomatal limitations, and photosynthesis

Stomata play a key role in regulating fluxes of water and carbon dioxide between plant and atmosphere. They regulate both plant growth and cycles of mass and energy. Therefore, much attention has been focused on principles of stomatal regulation and several regulatory mechanisms have been identified. Most research has centred on the stomatal responses to various indices of water status and carbon balance (Farquhar and Sharkey, 1982; Jones, 1998; Buckley *et al.*, 2003). Surprisingly little attention has been paid to the responses of *g*_s_ to temperature, even though it is one of the most variable environmental factors. A few previous studies suggested a dependence of *g*_s_ on temperature. However, these studies have often provided conflicting results. While some evidence suggested that *g*_s_ increased with increasing temperature (Schulze *et al.*, 1974; Lu *et al.*, 2000; Mott and Peak, 2010), other studies found that temperature had no effect on stomata (Teskey *et al.*, 1986; Sage and Sharkey, 1987; Cerasoli *et al.*, 2014; von Caemmerer and Evans, 2015), or that increased temperature triggered stomatal closure (Weston and Bauerle, 2007; Lahr *et al.*, 2015). One explanation for the conflicting results across these studies might be that the experiments were often conducted in uncontrolled environmental conditions in the field. The design of our experiment, in which the response of *g*_s_ to *T*_l_ was separated from the effect of VPD and all measurements were made under constant illumination, allowed us to separate the effect of temperature from the effects of other factors.

Our results conclusively demonstrated that there is a strong direct positive response of *g*_s_ to increasing *T*_l_ in two tree species. In well-watered trees, temperature and VPD had major effects on *g*_s_, as suggested by Freeden and Sage (1999). Elevated *C*_a_ caused a decline in *g*_s_ but did not fully mitigate increased stomatal opening in response to increased temperature. The increase in *g*_s_ with increased *T*_l_ was found in both species despite large differences in leaf morphology, xylem structure, and physiology. However, because of these differences, the magnitude of stomatal opening in response to *T*_l_ and closing in response to elevated [CO_2_], along with the effects on associated physiological processes (such as *E* and *A*), differed between the two species. The interplay between elevated *T*_l_, which increased *g*_s_, and elevated [CO_2_], which decreased *g*_s_, differed between the two species, suggesting that it could contribute to differences in behaviour among species in the predicted future climate.

The two experimental species stand at opposite ends of the range of mechanisms for stomatal adjustment of water loss. *E* in poplar continuously increased with increasing VPD, while *E* of loblolly pine remained the same over a large range of VPD within a given *T*_l_ and increased with increases in *T*_l_ (Fig. 2, Supplementary Fig. S2). These results suggest that *g*_s_ is regulated by more complex mechanisms than simply *E* (Mott and Parkhust, 1991), and that temperature changes affect the relationship between *E* and *g*_s_.

Leaf water potential declined with both increased temperature and increased VPD in both species (Fig. 5). Typically, *g*_s_ declines with a decline in water potential across a wide range of both iso- and anisohydric species (Klein, 2014). But in our study, despite a decline in water potential, *g*_s_ increased with temperature. Stomata may have opened with increasing temperature owing to, in part, changes in hydraulic conductivity. When temperature increases, the viscosity of water declines and mesophyll conductance (*g*_m_) increases, which may improve the supply of water to sites of evaporation and thus increase stomatal aperture (Cochard *et al.*, 2000; von Caemmerer and Evans, 2015). However, this increase was not great enough to prevent a decline in leaf water potential. Therefore, it was proposed that resistance to water vapour and heat transfer among sites of evaporation and guard cells, which induce differences in temperature and VPD at these sites, may also regulate stomatal opening in response to transpiration and *T*_l_ (Mott and Peak, 2013). The general increase in overall tree hydraulic conductance due to water viscosity may be further modified by temperature-dependent variability in tree xylem hydraulic conductance, which, due to differences in vascular traits, may contribute to differences in the responses of conifers and angiosperm trees (Wolf *et al.*, 2016). Changes in leaf *g*_m_ may be further paired with xylem resistance to embolism and the safety margin against cavitation, which is higher in conifers than in angiosperms (Choat *et al.*, 2012). Trees adjust their *g*_s_ to maximize CO_2_ uptake (resulting in higher *E*) but still protect xylem against excessive cavitation (Brodribb *et al.*, 2016). Loblolly pine strictly regulated transpiration such that it did not change with variation in VPD, thus protecting xylem against cavitation and maintaining a broad safety margin. However, when temperature increased, loblolly pine was not able to maintain this strict control over water loss, so *E* increased. This result may suggest that in the pine, overall resistance of the hydraulic pathway (including xylem and mesophyll resistance) significantly contributed to regulation of transpiration and that stomatal regulation was at least partly independent of *E*. In contrast, the broadleaf poplar exerted the same degree of stomatal control on *E* at all temperatures. The inability of loblolly pine to regulate *E* when temperature increases may negatively impact survival with climate change and may contribute to succession by angiosperm tree species (Carnicer *et al.*, 2013).

Apart from plant water status, other mechanisms known to regulate *g*_s_ are related to photosynthesis, to which stomata often present a large limitation. *L*_*s*_ in loblolly pine is usually lower than 65%. Higher *L*_s_ may occur but it is usually attributed to low soil water potential or low temperature (Teskey *et al.*, 1986; Sasek and Richardson, 1989; Ellsworth, 2000). In this study, when VPD was high, *L*_*s*_ of 78% was observed at 30°C (Table 1), indicating strong stomatal control of carbon gain in the range of temperature which is optimal for photosynthesis. With increasing *T*_l_, *L*_*s*_ declined. Therefore, photosynthesis of loblolly pine may partly benefit from the decline in *L*_s_ at increased temperature, even though the extremely high temperature will set biochemical limits to *A* and the resulting *A* may be the same or lower. In contrast to loblolly pine, *L*_s_ in poplar was unaffected by *T*_l_ and was generally lower than 20%. Low *L*_s_ in poplar in this study corresponded to low *L*_s_ in poplar observed previously; for example, *L*_s_ averaged 10% in two clones of *Populus* (Noormets and Sober, 2001). The lower *L*_*s*_ in poplar compared with loblolly pine may have been related to the ratio of *g*_s_ to *g*_m_. Although we did not measure *g*_m_, it is generally lower in conifers than in angiosperm trees (Flexas *et al.*, 2012), suggesting *L*_s_ should also be lower. However, because *L*_s_ was not lower, we speculate that the ratio of *g*_s_ to *g*_m_ also differed between the species. The high *A*_sat_ in poplar might be related to high *g*_s_/*g*_m_, which could support increased photosynthesis by increasing *C*_i_ and keeping [CO_2_] at the chloroplasts high. It could also increase nutrient acquisition through increased *E*, which would enhance photosynthetic capacity. *g*_m_ also increases with temperature in a wide range of species (von Caemmerer and Evans, 2015). However, this mechanism does not explain the increase in *g*_s_ at supra-optimal temperatures at which *A*_sat_ becomes low or negative.

Low *L*_s_ in poplar was linked to high *g*_s_, which results in low water use efficiency of photosynthesis. The advantage of low *L*_s_, which favours fast-growing species under an unlimited soil water supply, may jeopardize their existence during heat waves when high *E* depletes available soil water, resulting in increased drought stress, especially under initial conditions of low soil moisture. The effect of variable *L*_s_ was further demonstrated by the alteration of *C*_i_ in loblolly pine. Normally the ratio of *C*_i_:*C*_a_ is highly conserved (Liu and Teskey, 1995), as was observed in poplar when *C*_i_ consistently remained at ~300 μmol mol^−1^ at all temperatures (Fig. 3a). However, *C*_i_ in loblolly pine was highly variable, ranging from ~165 µmol mol^−1^ at 20°C to ~240 µmol mol^−1^ at 40°C (Fig. 3c), which corresponds with prior observations of high variability in *C*_i_ with changing environmental conditions in this species (Green and Mitchell, 1992).

### Evaporative cooling

Evaporation of water from the leaf surface can significantly lower *T*_l_ (Monteith, 1981; Jones, 1999). As long as stomata remain open, evaporative cooling can mitigate the negative effect of supra-optimal *T*_a_ on *A* during heat waves and can positively affect photosynthesis, yield, and plant survival (Lu *et al.*, 1994; Ameye *et al.*, 2012). Maintaining *T*_l_ through regulation of *E* to minimize stress at high *T*_a_ was theoretically suggested (Mahan and Upchurch, 1988) and observations in *Arabidopsis thaliana* indicated that plants regulate water loss and even adjust their architecture to achieve the best cooling effect (Crawford *et al.*, 2012). The magnitude of the cooling effect is often several degrees (Jones, 1999; Feller, 2006). In our study the maximum cooling, 9°C, was observed in poplar at high *T*_a_ and high VPD (Fig. 4). This rate of cooling lowered *T*_l_ from 49 to 40°C and positive *A* was observed at this extreme *T*_a_. In contrast to poplar, *g*_s_ of loblolly pine was roughly 10 times lower and therefore the maximum cooling effect was only 0.9°C. Consequently, at *T*_a_ of 49°C, poplar had positive *A* and loblolly pine did not. The cooling effect due to stomatal opening at high temperature (under well-watered conditions) is likely to be much more beneficial in species with high *g*_s_ than those with low *g*_s_.

Evaporative cooling may help plants survive heat waves, especially when the air is dry. However, this mechanism requires sufficient soil water supply, which relies on high soil water capacity and sufficient hydraulic conductivity. With a long-duration heat wave, high *E* may result in the depletion of soil water storage and plants will no longer be able to utilize this mechanism to minimize heat stress. This effect was observed in our study: only a very small cooling effect (1.1°C) was observed in drought-stressed trees (Fig. 4b). Nevertheless, evaporative cooling proved to have a significant effect on *A* and may play an important role in the diurnal regulation of *T*_l_ during short-duration heat waves. In addition to soil water availability, elevated [CO_2_] affects *g*_s_. Stomatal closure resulting from elevated [CO_2_] will to some degree counteract the opening effect of elevated temperature. Results of this study, demonstrating that stomata of poplar are more sensitive to [CO_2_] than stomata of loblolly pine, were similar to previous findings on broadleaf and conifer species in general (Medlyn *et al.*, 2001). Therefore, if stomata in broadleaf species close in response to future predicted increases in [CO_2_], the difference in the rate of evaporative cooling between broadleaf and conifer species may shrink.

### Relationships among *g*_s_, *C*_i_, and *A*

In both species we found that the positive relationship between *A*_sat_ and *g*_s_ observed at lower temperatures was not present at extremely high temperatures. The most obvious impairment occurred at a *T*_l_ >40°C, when *A*_sat_ became negative and yet the stomata remained open (Fig. 3). *C*_i_ at this temperature increased and approached the ambient [CO_2_] of 400 µmol mol^−1^. Under these conditions a reduction in *g*_s_ would be expected (Hashimoto *et al.*, 2006), but instead the stomata opened even more. These results do not imply that stomata do not react to *C*_i_. Rather, it appears that there was a direct stomatal response to supra-optimal temperature that overrode the response to *C*_i_.

Many models of *g*_s_ assume a fixed relationship between *A* and *g*_s_ regardless of temperature (Ball *et al.*, 1987; Leuning, 1995; Buckley *et al.*, 2003). These models have been widely used and, in a comparison with other models of *g*_s_, provided the best results (Way *et al.*, 2011). Our study also provided evidence of a stable relationship between *A*_sat_ and *g*_s_ at low temperatures (Fig. 3). However, that stability did not hold true at high temperature. As an extreme example, when *A*_sat_ became negative at temperatures over ~40°C, the ratio *A*:*C*_i_ also became negative in both species. In such a case, the Ball–Berry–Leuning model, which uses that ratio to predict *g*_s_, would provide negative values of *g*_s_. Correctly predicting *g*_s_ from photosynthesis and *vice versa*, especially at extreme temperatures during heat waves, will require detailed study of the interplay among *A*, *C*_i_, VPD, *T*_l_, and possibly other factors driving stomatal regulation, which, when applied simultaneously, can have complex effects (Merilo *et al.*, 2014).

## Conclusions

We conclude that *T*_l_ has a direct effect on stomatal opening in the two tree species we examined. For accurate predictions of *g*_s_ and plant water use this temperature dependency should be taken into account, especially at high temperatures. Elevated [CO_2_] reduced *g*_s_ of both species but general trends of increasing *g*_s_ with increasing *T*_l_ remained similar regardless of [CO_2_]. Along with changes in *g*_s_*, T*_l_ also affected *L*_s_ to photosynthesis, *C*_i_, and corresponding *A*. *A* became negative in both species at extremely high *T*_l_. However, the effect of evaporative cooling, which lowered *T*_l_ in the rapidly transpiring poplar, significantly increased *A*. *g*_s_ was decoupled from *A* at high *T*_l_ in both species, which is an indication that substantial changes are likely in gas exchange physiology at high temperatures. Further research should focus on verifying results of this laboratory study in the field, as well as discovering the principles of temperature dependency of stomatal regulation and implementing temperature functions into the models of *g*_s_.

## Supplementary data

Supplementary data are available at *JXB* online.

Fig. S1. *G*_s_ of poplar and loblolly pine and its dependence on *T*_a_ and VPD.

Fig. S2. *E* and *C*_i_ of poplar and loblolly pine and their dependence on *T*_a_ and VPD.

Fig. S3. *A*_sat_ of poplar and loblolly pine and its dependence on *g*_s_ at *T*_a_ 20–49°C.

Table S1. Regression equations and parameters of models used in Figs 1–5.

## Supplementary Material

Supplementary_Figures_S1_S3_table_S1

## Abbreviations:

Ψ_P_: pre-light water potential (Pa)
*A*: rate of net photosynthesis (μmol m^−2^ s^−1^)
*A*_sat_: light-saturated net photosynthesis (μmol m^−2^ s^−1^)
*C*_a_: atmospheric concentration of CO_2_ (μmol mol^−1^)
*C*_i_: intercellular concentration of CO_2_ (μmol mol^−1^)
*E*: rate of transpiration (mol H_2_O m^−2^ s^−1^)
*g*_m_: mesophyll conductance (mol m^−2^ s^−1^)
*g*_s_: stomatal conductance (mol m^−2^ s^−1^)
*J*_max_: maximum rate of photosynthetic electron transport (μmol m^−2^ s^−1^)
*L*_s_: stomatal limitation to photosynthesis (%)
PAR: photosynthetically active radiation
*R*_*d*_*: day respiration (μmol m^−2^ s^−1^)
*T*_a_: air temperature (°C)
*T*_l_: leaf temperature (°C)
*V*c_max_: maximum rate of Rubisco carboxylation (μmol m^−2^ s^−1^)
VPD: vapour pressure difference (Pa)
*V*_TPU_: maximum rate of triose-phosphate utilization (μmol m^−2^ s^−1^).
