# Supplementary material for: Increase in leaf temperature opens stomata and decouples net photosynthesis from stomatal conductance in Pinus taeda and Populus deltoides x nigra
Source: J Exp Bot. 2017 Mar 6;68(7):1757–67. doi: 10.1093/jxb/erx052 (PMC5444456; doi:10.1093/jxb/erx052)
Supplement: Supplementary_Figures_S1_S3_table_S1 [file erx052_suppl_Supplementary_Figures_S1_S3_table_S1.pdf]

Supplementary Figure S1

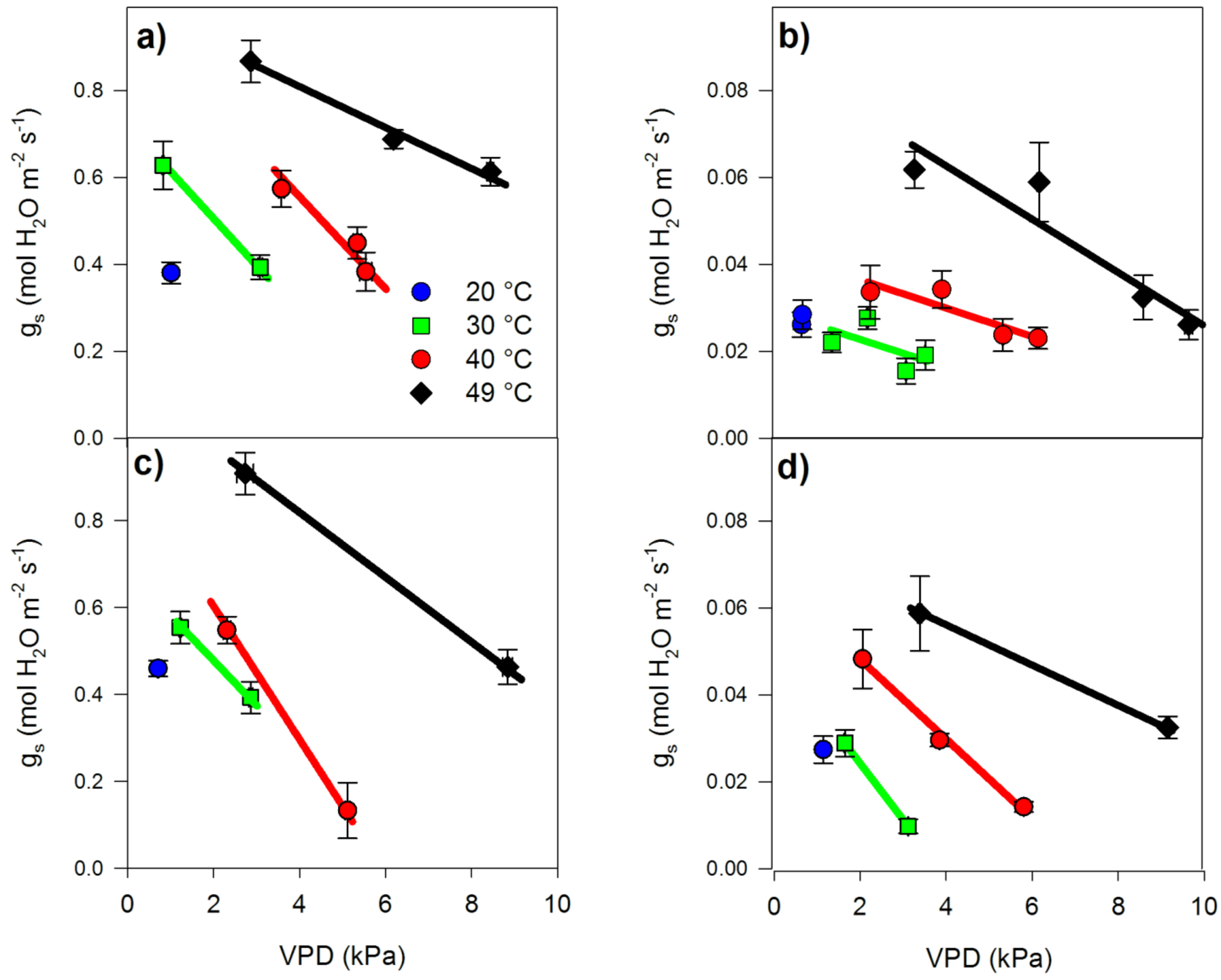

Supplementary Figure S1. Stomatal conductance (g_s) of poplar (left panels) and loblolly pine (right panels) and its dependence on air temperature and vapor pressure deficit (*VPD*). Plants were measured in high soil moisture conditions and ambient [CO_2] (panels a and b) or elevated [CO_2] (panels c and d). Error bars indicate standard error of the mean (n = 6). Linear regression was used to fit the data at the same temperatures.

Supplementary Figure S2

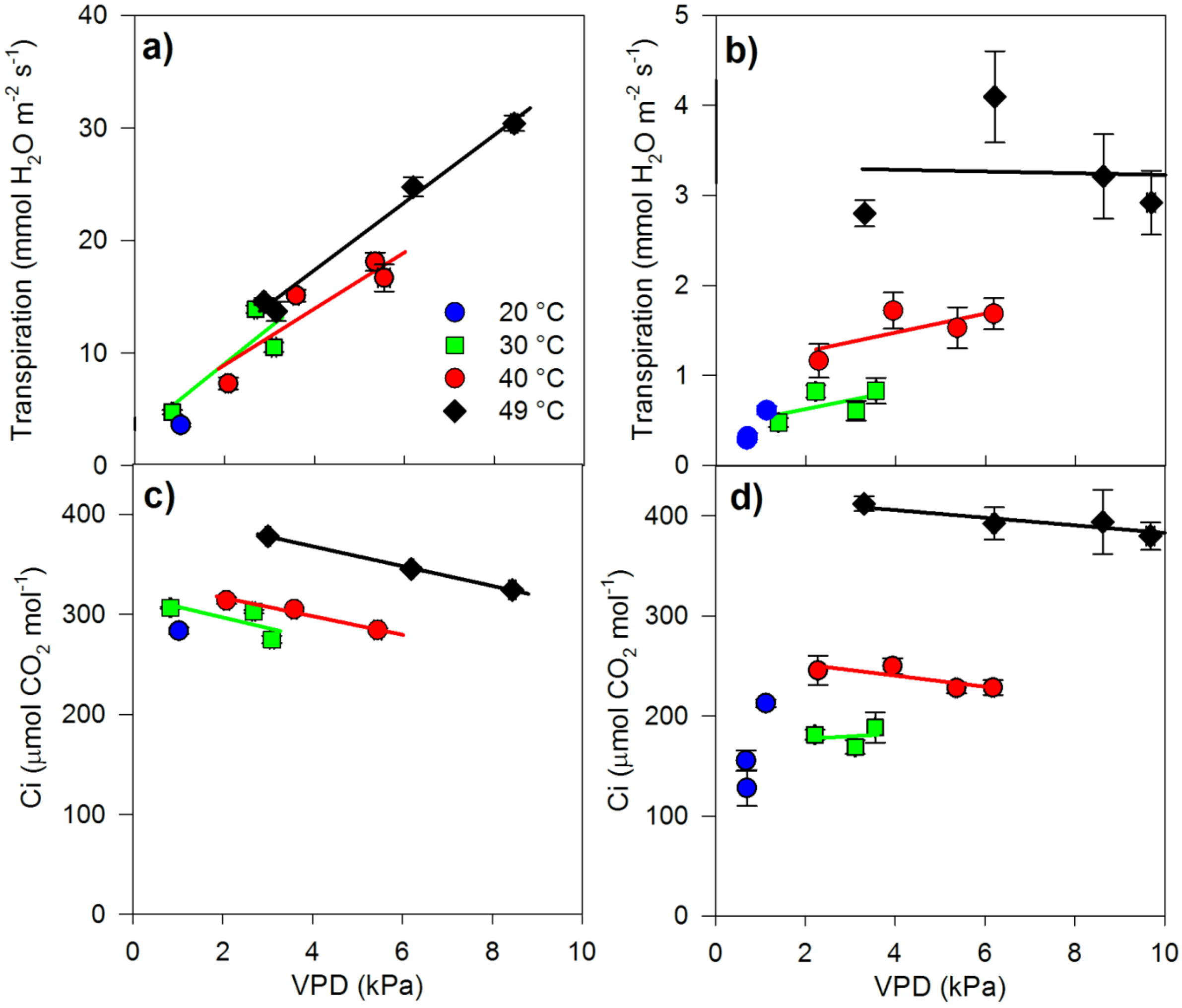

Supplementary Figure S2. Transpiration of poplar (a) and loblolly pine (b) and intercellular [CO_2] (C_i) of poplar (c) and loblolly pine (d) and their dependence on air temperature and vapor pressure deficit (*VPD*). Plants were measured in high soil moisture conditions and ambient [CO_2]. Error bars indicate standard error of the mean (n = 6). Linear regression was used to fit the data at the same temperatures.

Supplementary Figure S3

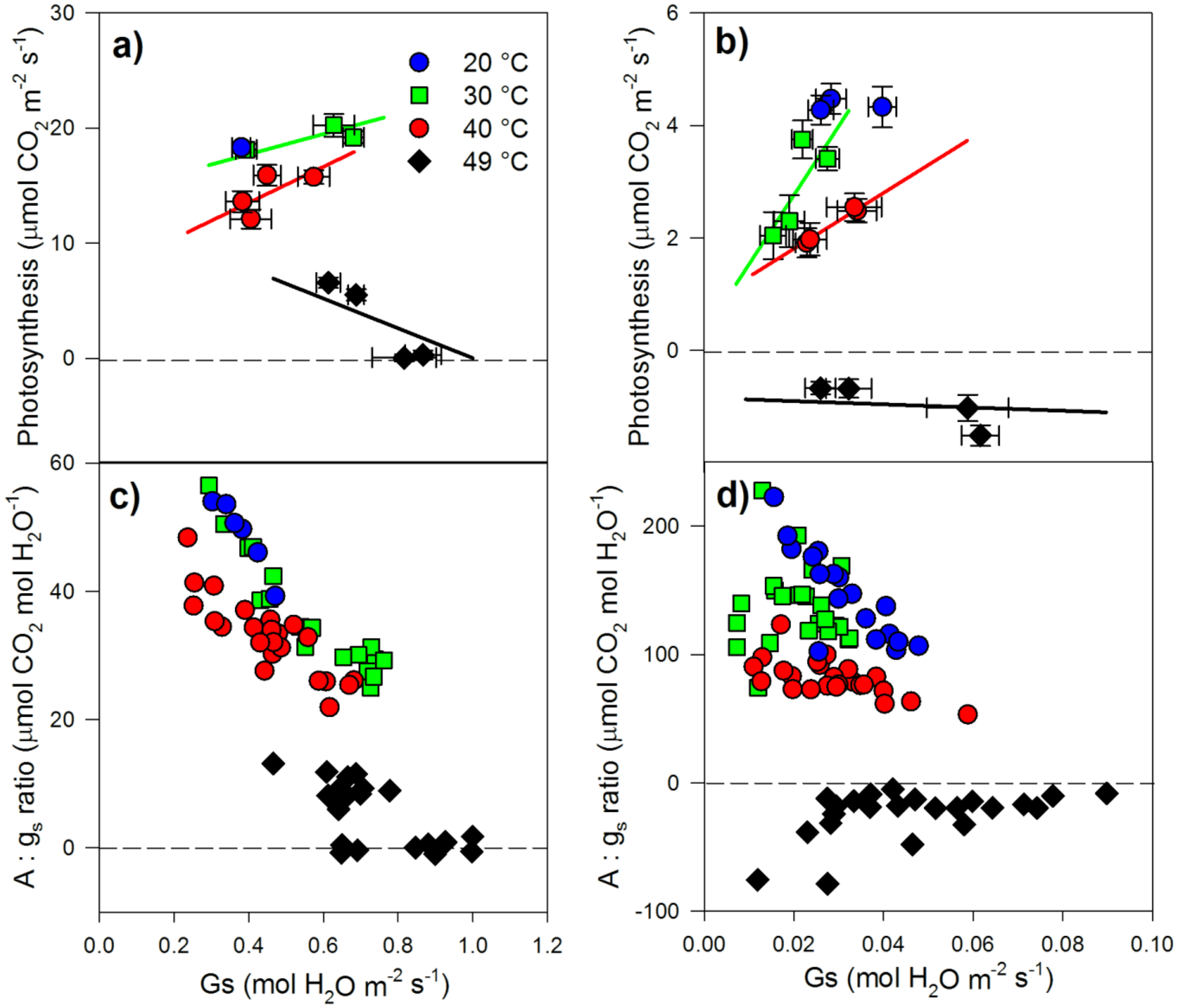

Supplementary Figure S3. Photosynthesis of poplar (a) and loblolly pine (b) and its dependence on stomatal conductance (g_s) at air temperatures 20 – 49 °C. Ratio of net photosynthesis (A) to stomatal conductance at various g_s at air temperatures 20 – 49 °C in poplar (c) and loblolly pine (d). Plants were measured in high soil moisture conditions and ambient [CO_2]. Error bars indicate standard error of the mean (n = 6).

Supplementary Table S1. Regression equations and parameters of models used in Figures 1 - 5.

Figure	Panel	Formula	y_0	a	b	c	d	x	y	p	R^2
1	a	$f = y_0+a*x+b*y$	-0.0401	0.0243	-7.67E-05			T_l	VPD	**<0.0001**	0.75
1	b	$f = y_0+a*x+b*y$	0.0375	0.0217	-0.0001			T_l	VPD	**<0.0001**	0.45
1	c	$f = y_0+a*x+b*y$	-0.0076	0.0008	-1.71E-06			T_l	VPD	**0.0498**	0.17
1	d	$f = y_0+a*x^c+b*y$	0.0333	5.25E-10	-5.31E-06	4.713		T_l	VPD	**<0.0001**	0.52
1	e	$f = y_0+a*x^c+b*y$	0.0268	4.99E-07	-6.19E-06	2.956		T_l	VPD	**<0.0001**	0.43
1	f	$f = y_0+a*x^c+b*y$	0.0054	9.54E-11	-7.74E-07	4.653		T_l	VPD	**<0.0001**	0.37
2	a	$f = y_0+a*x+b*y$	-4.4023	0.0969	0.0058			T_l	VPD	**<0.0001**	0.86
2	b	$f = y_0+a*x+b*y$	-1.7551	0.0846	7.00E-05			T_l	VPD	**<0.0001**	0.68
3	a	$f = y_0+a*x+b*y$	262.7	2.7284	-0.0132			T_l	VPD	**<0.0001**	0.39
3	b	$f = y_0+a*x+b*y+c*x^2+d*y^2$	-9.0903	2.0586	12.9	-0.0449	-4.53	T_l	g_s	**<0.0001**	0.91
3	c	$f = y_0+a*x+b*y$	67.6	4.6863	0.0063			T_l	VPD	**<0.0001**	0.56
3	d	$f = y_0+a*x+b*y+c*x^2+d*y^2$	-1.9285	0.3262	116.6	-0.0072	-1143	T_l	g_s	**<0.0001**	0.89
4	a	$f = y_0+a*x+b*y$	-0.5997	0.0389	-0.0015			T_a	VPD	**<0.0001**	0.91
4	b	$f = y_0+a*x+b*y$	-1.3451	0.0101	-3.41E-05			T_a	VPD	0.8757	0.01
4	c	$f = y_0+a*x+b*y$	-0.809	-0.0015	-3.04E-05			T_a	VPD	0.4092	0.02
5	a	$f = y_0+a*x+b*y$	0.3331	-0.0267	-6.42E-05			T_l	VPD	**<0.0001**	0.83
5	b	$f = y_0+a*x+b*y$	-0.5113	-0.0146	-4.92E-05			T_l	VPD	**0.0032**	0.24
5	c	$f = y_0+a*x+b*y$	0.2208	-0.0294	-8.79E-05			T_l	VPD	**<0.0001**	0.74
5	d	$f = y_0+a*x+b*y$	-1.4747	0.0122	-0.0001			T_l	VPD	**<0.0001**	0.56
